# An Analysis of Publicly Available National Health Service Information Leaflets for Patients With Shoulder Osteoarthritis

**DOI:** 10.1002/msc.70028

**Published:** 2024-12-18

**Authors:** Stacey Lalande, Maria Moffatt, Toby Smith, Vrinda Aggarwal, Chris Littlewood

**Affiliations:** ^1^ Airedale NHS Foundation Trust Keighley UK; ^2^ University of Liverpool Liverpool UK; ^3^ University of Warwick Coventry UK; ^4^ Edge Hill University Ormskirk UK; ^5^ University of Salford Salford UK

**Keywords:** management, patient information, physiotherapy, shoulder osteoarthritis

## Abstract

**Background:**

Shoulder osteoarthritis is a common cause of pain, disability and difficulty sleeping. Patient information leaflets are produced by NHS Trusts with the aim of informing patients about their diagnosis and available treatment options.

**Objectives:**

The aim of this study was to identify and describe the non‐surgical management of people with shoulder osteoarthritis according to publicly available information leaflets produced by NHS Trusts.

**Methods:**

One reviewer undertook an electronic search using Google to identify publicly available patient‐facing information leaflets (PIL) produced by NHS Trusts which detailed non‐surgical management strategies for people with shoulder osteoarthritis. Relevant data were extracted by one reviewer and verified by two reviewers.

**Results:**

Seventeen PILs from 17 different UK NHS Trusts were identified ranging from December 2016 to February 2024. Information provided in the PIL varied, with topics including general osteoarthritis management, exercise, analgesia, injections, and surgical indications. No PIL covered all areas recommended in the NICE Osteoarthritis Management guidelines.

**Conclusion:**

PILs developed and published by NHS Trusts are variable in content and do not fully reflect current clinical guidelines. High‐quality research to inform consistent, clinically, and cost‐effective treatment pathways, including information provision, for patients with shoulder osteoarthritis is needed.

## 1 Introduction

Shoulder osteoarthritis (OA) is a common cause of shoulder pain, disability and difficulty sleeping (Stanborough, Bestic, and Peterson 2022). It is thought that up to 20% of people over 65 may have radiographic evidence of shoulder OA (Ansok and Muh 2018). The health and economic burden of OA is expected to significantly increase by 2050 (Steinmetz et al. 2023) and thus the need for effective strategies to manage people with OA is critical (Hunter and Bierma‐Zeinstra 2019). Current UK clinical guidelines recommend that people with OA receive therapeutic exercise, weight management, advice and information about OA and pharmacological treatments (National Institute for Health and Care Excellence 2022).

Online resources, including websites and patient information leaflets (PIL), are developed, and produced by NHS Trusts to complement face‐to‐face services with the aim of informing patients about their diagnosis and available treatment options (Rohun, May, and Littlewood. 2021). These resources are an important means of complementing face‐to‐face services and thus it is important that they are accurate, comprehensive, reflective of current clinical guidelines and useful to patients (May et al. 2022).

Hence, as a component of a multi‐method programme of research aiming to optimise non‐surgical treatment pathways for people with shoulder OA, the aim of this study was to identify and describe the non‐surgical management of people with shoulder OA as described by publicly available PIL produced by NHS Trusts.

## 2 Methods

Ethical approval was not required for this review of publicly available information.

One reviewer (VA) undertook electronic searches of Google for publicly available patient‐facing information leaflets (PIL) from websites of UK NHS Trusts. The following search terms were used:

1. NHS, Shoulder, Osteoarthritis
2. NHS, Glenohumeral, Osteoarthritis

Searching continued until one full‐page of search results returned no relevant PIL. Results were recorded in Microsoft Excel. Any queries around the suitability of inclusion were resolved through discussion between the authors (VA, SL and CL).

## 3 Inclusion Criteria

Any PIL hosted on the official website of a UK NHS Trust that included information relating to the diagnosis and non‐surgical management of people with shoulder OA was included. Specifically, PILs relating to glenohumeral OA were retrieved, but for the purpose of reporting we use the term shoulder OA.

## 4 Exclusion Criteria

PILs from sources which were not UK NHS trusts were excluded, including those from non‐NHS organisations treating NHS patients.

### 4.1 Data Extraction

One reviewer (VA) extracted data from each PIL onto a pre‐developed Microsoft Excel data extraction form. These data were verified by two additional reviewers (SL and CL). Any queries around the suitability of inclusion were resolved through discussion between reviewers. Table 1 details the data extraction categories.

**TABLE 1:** Data extraction categories and detailed description.

Data extracted	Description
NHS trust	Which NHS trust published the leaflet
Date of publication	If available, the date which the information had been published by the trust.
General advice	Did the PIL include general advice on managing OA and if it did what was this advice?
Lifestyle choices	Did the PIL include general advice around lifestyle choices, such as smoking and obesity, and if it did what was this advice?
Exercise recommendations	Did the PIL include advice around exercise if it did what was this?
Strength	If the PIL included advice to perform strengthening exercises what were these?
Range of movement	If the PIL included advice to perform range of movement exercises, what were these?
Other	If the PIL included other exercises advice, for example cardiovascular exercise, what was this?
Medication	Did the PIL include advice around medication? If so, what was this advice?
Injections	Did the PIL include advice around injections? If so, what was this?
Other	If other information was included outside of the above domains, this was recorded here.

### 4.2 Statistical Analysis

Descriptive statistics (frequencies/percentages) were used to summarise the extracted data. All analyses were performed on Microsoft Excel.

## 5 Results

**FIGURE 1:**
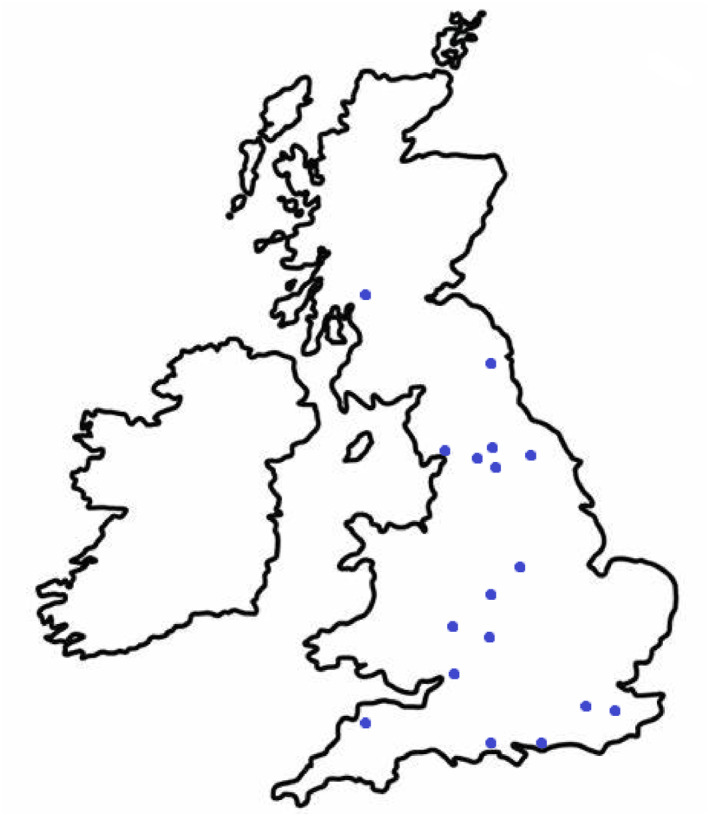
Geographical distribution of NHS Trusts included.

Sixteen PILs were identified using Search Terms 1 and 12 PILs were identified using Search Term 2. After removal of duplicates, 17 PILs from 17 different UK NHS Trusts were included. Of 17, 13 (76%) reported dates of publication. These ranged from December 2016 to February 2024. Geographical distribution of the NHS Trusts included can be seen in Figure 1 below:

### 5.1 Advice Provided

**FIGURE 2:**
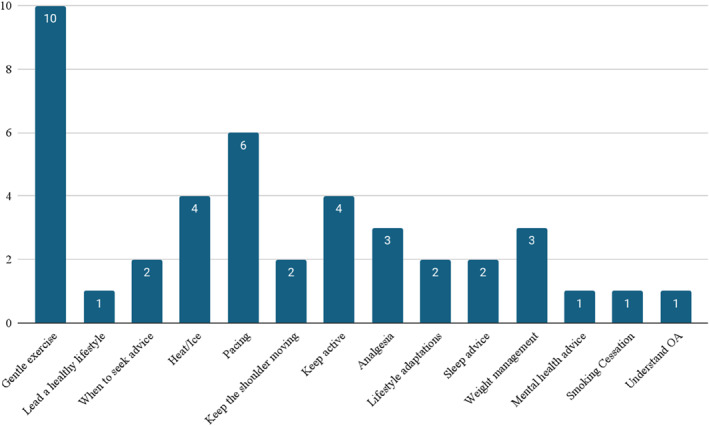
General advice on living with shoulder OA included in PILs.

Of the 17 PILs included, 16 (94%) provided general advice on living with shoulder OA. A breakdown of advice given is presented in Figure 2.

### 5.2 Lifestyle Choices

Nine PILs (53%) included specific advice on modifiable health behaviours. Five (29%) encouraged weight loss when appropriate, four (24%) encouraged general exercise, one (6%) encouraged a balanced diet, three (18%) promoted smoking cessation and one (6%) discussed stress management.

### 5.3 Exercise

**FIGURE 3:**
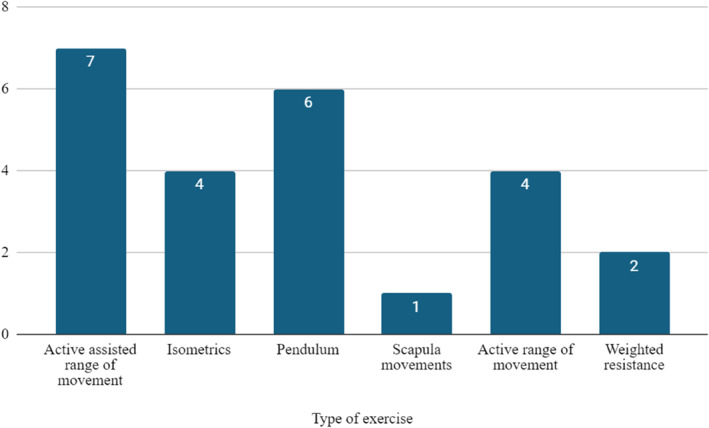
Specific shoulder exercises are recommended in PILs.

Fourteen PILs (82%) encouraged some form of shoulder exercise. 6 (35%) PILs recommended strengthening exercise, 11 (65%) recommended range of movement shoulder exercises, and 8 (47%) encouraged general cardiovascular exercise. The specific types of shoulder exercises recommended are illustrated in Figure 3.

### 5.4 Medication

Ten PILs (59%) included medication advice. All provided advice on over‐the‐counter analgesia. One PIL (6%) provided advice on prescription analgesia and one PIL (6%) provided advice on additional supplements.

Of the 10 providing advice on over‐the‐counter analgesia, paracetamol was recommended in five PILs, oral anti‐inflammatory medication in six PILs, and topical anti‐inflammatory medication once.

### 5.5 Injections

Nine PILs (53%) discussed joint injections in the management of shoulder OA pain. Eight of these referred to steroid injections of an unspecified location, whilst the remaining one referred to an unspecified injection in terms of the injectable solution or the anatomic location.

### 5.6 Other Advice

**FIGURE 4:**
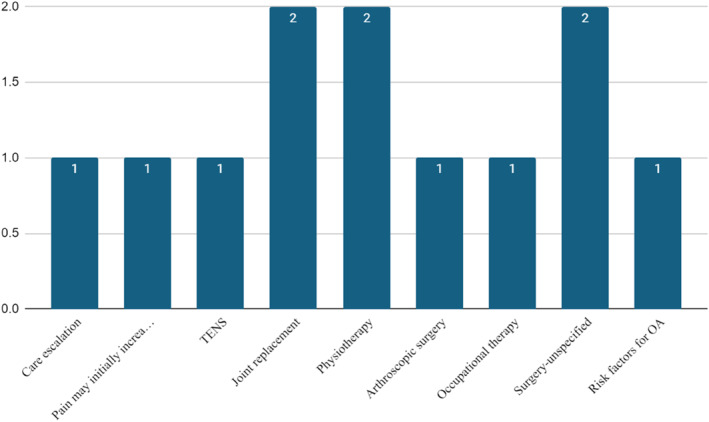
Additional advice in PIL.

Thirteen PILs (76%) provided additional advice not previously discussed. As summarised in Figure 4, this included five (29%) providing further non‐surgical intervention advice, which included one (6%) discussing when to escalate care, one (6%) advising that pain may initially increase when starting to exercise and advice around risk factors in one (6%). There was also signposting to other information internally (same NHS Trust *n* = 1) and national charities (vs. Arthritis; *n* = 1).

## 6 Discussion

This review of UK NHS Trust's PILs in the public domain suggests that, in‐general, the information currently provided is inconsistent and does not align with NICE guidelines for the management of people with OA (National Institute for Health and Care Excellence 2022; National Institute for Health and Clinical Excellence 2016).

Exercise is a core treatment recommendation for all people with osteoarthritis (National Institute for Health and Care Excellence 2022). It is recommended that exercise should be therapeutic and tailored to the needs of individuals. It is recommended that it should be accompanied with the advice that exercise may initially cause pain or discomfort, but long‐term adherence to an exercise plan will offer benefits with reduced pain and improved function (National Institute for Health and Care Excellence 2022). Reassuringly, 14 (82%) PILs included shoulder exercises, with 8 (47%) encouraging general cardiovascular exercise. Only one (6%) mentioned that pain may initially increase when exercising. It has been shown that both surgeons and physiotherapists have mixed views on the role of exercise in the management of shoulder OA (Kane et al. 2024). However, both agree that as the radiographic severity of shoulder OA progresses, the likely benefits of exercise decrease (Kane et al. 2024). In most PILs included in this study, it was unclear who the primary authors were, for example physiotherapist, orthopaedic surgeon, patients and the inconsistency might be reflective of the individual views of the authors, although this cannot be substantiated. However, to date, research in this area of shoulder osteoarthritis has not focused on developing and testing acceptable and effective exercise programmes. A feasibility trial by Larsen et al. (2022) reported that progressive exercise was safe and may relieve pain and improve function and range of movement in people with shoulder OA but this was a single group, non‐randomised study, limited to 20 participants. The therapeutic exercise recommendation from current clinical guidelines is based on research which has been conducted on those with OA of the hip or knee, and there is a dearth of evidence relating to people with shoulder OA.

Ten of the 17 (59%) PILs recommended analgesia for people with shoulder OA, but this did not include the recommendation to use analgesia to support therapeutic exercise (National Institute for Health and Care Excellence 2022). Paracetamol was recommended as a first‐line analgesic in five (29%) PILs; oral non‐steroidal anti‐inflammatory medications in six PILs (35%); and topical non‐steroidal anti‐inflammatory medications in one PIL (6%). This conflicted with the guidance that topical NSAIDs should be offered first and oral only when topical proves to be ineffective or unsuitable (National Institute for Health and Care Excellence 2022). Steroid injections were also discussed in PILs relatively frequently, with nine (53%) PILs including information on these. Intra‐articular steroid injections are recommended for short term pain relief when other pharmacological treatments are ineffective or to support therapeutic exercise (National Institute for Health and Care Excellence 2022). There are no high‐quality studies which have explored the use of intra‐articular steroid injections for treating patients with shoulder OA. The use of steroid injections in shoulder OA is based on its effects on the synovium and surrounding tissue, as well as its clinical efficacy in other joints, especially the knee (Gross et al. 2013). Therefore, the evidence‐base behind this recommendation may also be questioned for this particular joint, over more general OA management.

It has previously been reported that reliable information about OA is hard for patients to identify, but that good quality information beyond simply providing information about OA can help to reduce the impact of OA (Wallis et al. 2019). Addressing patients' information needs can help to address the negative or mistaken language and beliefs about their OA through education. It is not known if the 2022 NICE OA guidelines (National Institute for Health and Care Excellence 2022) are relevant or effective for the management of symptoms in those with shoulder OA, due to a reliance on evidence from studies investigating people with hip and knee OA. However, it is apparent that PILs produced by NHS Trusts, on the whole, do not provide advice which is wholly in‐line with this national guidance.

### 6.1 Strengths and Limitations

This is the first study to map non‐surgical management practice recommendations for people diagnosed with shoulder OA based on publicly available PIL. It is, however, a snapshot of NHS Trusts who provided PILs online and therefore may be a selective sample, not representative of wider practice.

## 7 Conclusion

PILs developed and published by NHS Trusts are variable in content and do not fully reflect current clinical guidelines. High‐quality research to inform consistent, clinically and cost‐effective treatment pathways, including information provision, for patients with shoulder osteoarthritis is needed.

## Author Contribution

C.L. and M.M. conceived the study. C.L., S.L. and V.A. developed the search strategy and V.A. undertook the search. V.A. extracted the data, and this was verified by C.L. and S.L. S.L. drafted the manuscript, and all authors reviewed and approved the manuscript.

## Ethics Statement

Ethical approval was not required for this review of publicly available information.

## Conflicts of Interest

The authors declare no conflicts of interest.

## Data Availability

The data that support the findings of this study are available from the corresponding author upon reasonable request.
